# Metabolic and endocrine connections of 17-hydroxypregnenolone in polycystic ovary syndrome women

**DOI:** 10.1530/EC-17-0151

**Published:** 2017-08-07

**Authors:** Sebastião Freitas de Medeiros, Cinthia Marenza Ormond, Matheus Antônio Souto de Medeiros, Nayara de Souza Santos, Camila Regis Banhara, Márcia Marly Winck Yamamoto

**Affiliations:** 1 Department of Gynecology and ObstetricsMedical School, Federal University of Mato Grosso, Cuiabá, Mato Grosso, Brazil; 2 Tropical Institute of Reproductive Medicine and MenopauseCuiabá, Mato Grosso, Brazil

**Keywords:** polycystic ovary syndrome, hyperandrogenism, adrenal steroidogenesis, 17-hydroxypregnenolone, androgens, hormone precursor

## Abstract

**Objective:**

To examine the anthropometric, and metabolic connections of 17-hydroxypregnenolone in the normo- and hyperandrogenemic polycystic ovary syndrome phenotypes.

**Materials and methods:**

This cohort study was conducted at the Julio Muller University Hospital, Cuiabá, Brazil, between January 2014 and July 2016, and 91 normal cycling healthy women, 46 normoandrogenemic and 147 hyperandrogenemic, patients with polycystic ovary syndrome (PCOS) were enrolled according to the Rotterdam criteria. Several anthropometric, biochemical and hormonal parameters were properly verified and correlated with 17-hydroxypregnenolone (17-OHPE) concentrations.

**Results:**

17-OHPE was higher in hyperandrogenemic PCOS than in normoandrogenemic PCOS and in control groups (*P* = 0.032 and *P* < 0.001, respectively). In healthy controls, 17-OHPE was positively associated with glucose, free estrogen index, DHEAS and negatively associated with compounds S. In normoandrogenemic PCOS patients, 17-OHPE presented positive correlations with VAI, LAP, cortisol, insulin and HOMA-IR. In the hyperandrogenemic group, 17-OHPE presented significant negative correlations with most anthropometric parameters, HOMA-IR, HOMA %B, estradiol, free estrogen index (FEI), C-peptide, and TG levels and positive correlations with HOMA-S and high-density lipoprotein cholesterol (HDL-C), sex-hormone binding globulin (SHBG), androstenedione (A4) and dehydroepiandrosterone (DHEA). Regarding hyperandrogenemic PCOS, and using a stepwise multiple regression, only HOMA-S and WHR were retained in the model (*R*^2^ = 0.294, *P* < 0.001).

**Conclusion:**

17-OHPE exhibited different relationships with anthropometric, and biochemical parameters in PCOS patients, depending on the androgen levels. In PCOS subjects with high androgen concentrations, 17-OHPE was negatively associated with most anthropometric parameters, particularly with those used as markers of adipose tissue dysfunction and frequently employed as predictors of cardiovascular disease risk; otherwise, 17-OHPE was positively associated with HDL-C and HOMA-S in this patients. Future studies are required to evaluate the clinical implications of these novel findings.

## Introduction

After standardization of the diagnostic criteria for polycystic ovary syndrome (PCOS) the hyperandrogenemic phenotype has been found in nearly 80% of patients (1, 2). These hyperandrogenemic patients may have their androgen synthetized by ovaries, adrenal gland, or both. It is estimated that between 20% and 30% of these patients have the adrenal as principal source of androgens (3, 4). In the adrenal ∆5 androgen synthesis pathway, the microsomal enzyme P450c17 mediates both 17-hydroxylase and 17, 20 lyase activities, with greater 17-hydroxylase activity in the adrenal fasciculate layer and equivalent activities in the reticular layer (5). In the ∆4 pathways, 17-hydroxylase activity is similar to that presented in ∆5, but the 17, 20 lyase activity is minimal in humans (6). The 17-hydroxylase induces rapid conversion of substrate pregnenolone (PE) and progesterone (P4) into intermediate precursors 17-hydroxypregnenolone (17-OHPE) and 17**-**hydroxyprogesterone (17-OHP4), respectively. The 17, 20 lyase mediates the conversion of 17-OHPE and 17-OHP4 into dehydroepiandrosterone (DHEA) and androstenedione (A4), respectively (5, 7). Other adrenal enzymes, 3β-hydroxysteroide dehydrogenase II (3β-HSD II) and sulfotransferase (SULT2 A1) drive these precursors toward the synthesis of ∆4 steroids and dehydroepiandrosterone sulfate (DHEAS), respectively (7).

After exclusion of other hyperandrogenemic conditions such as hyperprolactinemia, thyroid dysfunction and the classic steroidogenic enzyme deficiences, small defects or imbalance in the enzymatic activities seen in some PCOS patients may account for the biochemical hyperandrogenism (4, 8, 9). Hyperandrogenemic PCOS patients may present decreased 3β-HSD II activity with a lower conversion rate of ∆5 into ∆4-hydroxysteroids resulting in higher amount of substrates for androgen synthesis (10). In fact, hyperandrogenemic PCOS patients may have different levels of 3β-HSD II activity in different adrenal cells (11), presenting higher 3β-HSD II activity in the conversion of DHEA into A4 than in the conversion of 17-OHPE into 17-OHP4 (4, 11). Furthermore, PCOS patients commonly have dysregulation of the P450c17α enzymatic complex with upregulated 17, 20 lyase activity (12). However, the principal abnormality in P450c 17α in PCOS seems to be an increase in the activity of 17-hydroxylase relative to that of 17,20 lyase in the ∆4 pathway (8), with increased synthesis of 17-OHPE and DHEA (5, 13).

In the clinical setting, DHEA and DHEAS are considered as principal markers of adrenal androgen synthesis and little attention has been given to their precursor 17-OHPE in PCOS subjects. Testing for adrenal androgens is useful for differential diagnosis of PCOS and adult congenital adrenal hyperplasia (CAH), which may be clinically indistinguishable. Several other androgens and/or precursors can be raised when total testosterone (T) is normal in PCOS (14). It is possible that patients with normal T levels and high A4, or any other androgen precursor, have similar risk for metabolic disease as those women with a high T concentrations (15). Higher concentrations of 17-OHPE levels, commonly found in premature pubarche and adolescent females with signs of hyperandrogenism, are frequently associated with abnormal activities of both P450c 17α and 3β-HSD II enzymes (10, 13). The concentrations of 17-OHPE in PCOS patients have not been explored after its diagnosis standardization and the exact clinical implications of all adrenal precursor-androgens (APA) remain unknown. The present study aims to examine the connection of the ∆5 androgen precursor 17-OHPE with anthropometric markers of adipocyte dysfunctions, other androgens, and markers of dysmetabolism in PCOS patients either with normal or high androgens concentrations in blood.

## Materials and methods

The current study, approved by the Research Ethics Committee of the Federal University of Mato Grosso, was conducted at the Julio Muller University Hospital and the Tropical Institute of Reproductive Medicine, Cuiabá, MT, Brazil, between January 2014 and July 2016. Written informed consent was obtained from each woman. Using accessibility sampling, the sample consisted of 193 PCOS patients and 91 healthy normal cycling women in whom 17-OHPE concentrations were measured. Patients were excluded if they have used sex steroid, or insulin-sensitizing drugs over the last 6 months. In addition, patients with thyroxin-stimulating hormone (TSH) levels ≥4.2 µIU/mL, prolactin >1086 pmol/L, and 17-OHP4 ≥6 nmol/L were excluded from analysis (9, 16). Classic 3β-HSD II, and 11β-hydroxylase deficiencies were excluded in the following cases: 17-OHPE <15 nmol/L, and 11-deoxycortisol (11-DOC, compound S) <0.2 µmol/L) (4, 17).

Biochemical hyperandrogenism (hyperandrogenemic PCOS) was defined by total testosterone (T) ≥2.1 nmol/L, DHEAS ≥6.7 µmol/L, A4 ≥8.6 nmol/L and free androgen index (FAI) ≥6 (4, 18, 19). Polycystic ovary morphology was defined according to previous recommendations (20). Obesity was defined as body mass index (BMI) ≥30 (kg/m^2^). HOMA-IR, HOMA %B, HOMA-S were calculated using a free online program (21). HOMA-IR cut off was set ≥2.7 (22).

The subjects were weighed on an electronic scale, and height was measured using a Harpender stadiometer (Holtain Limited, Crymych, Dyfed, UK). The waist circumference (WC) was measured at the midway point between the lower rib margin and the iliac crest, and the hip was measured at the widest circumference (location of the greater trochanters). BMI was calculated as body weight (kg/height (m)^2^). Lean body mass (LBM) was calculated using the James equation: (1.07 × weight (kg)) − 148 × (weight^2^/(100 × height (m))^2^ (23). Fat mass (FM) was calculated as: body weight − LBM. Abdominal adiposity was estimated using the conicity index (C index): WC (m)/(0.109 × square root of body weight (kg)/height (m)) (24). The visceral adiposity index (VAI) was estimated using the equation: WC/(36.58 + (1.89 × BMI)) × (TG/0.81) × (1.52/HDL-C) (25). Lipid accumulation product (LAP) was calculated as (WC (cm) − 58) × (TG (mmol) as established) for women (26).

Blood samples were obtained between 07:00 and 09:00 h by venupuncture after a 10–12 h fast. All patients with regular cycles were tested in the early follicular phase of the menstrual cycle (days 3–5 of the cycle). Patients with infrequent menses or amenorrhea had their blood collected at any time provided the progesterone was less than 6.4 nmol/L. Triglycerides (TG), high-density lipoprotein cholesterol (HDL-C), and total cholesterol (TC) levels were measured using an enzymatic assay (Wiener Laboratories, Rosário, Argentina). Low-density lipoprotein cholesterol (LDL-C) was calculated as TC-(HDL-C + TG/5) (27). Glucose concentration was analyzed using the glucose oxidase technique (Beckman Glucose Analyses, Fullerton, CA, USA).

Serum P4 was measured using a chemiluminescence assay (Advia Centaur, Siemens Healthcare Diagnostics, UK) with a sensitivity of 0.67 nmol/L, coefficients of intra- and inter-assay variation were lower than 12% and 4%, respectively. Serum thyroid stimulating hormone (TSH), estradiol (E2), prolactin (PRL), sex-hormone binding globulin (SHBG), total testosterone (T), DHEA and free thyroxin (FT4) were measured with an electrochemiluminescence assay (Elecsys 1010, Roche Diagnostics GmbH). The intra- and inter-assay coefficients of variation were lower than 10% for all analytes. A4, DHEAS, cortisol (F), and insulin were measured using a chemiluminescence assay with a sensitivity of 1.0 nmol/L, 0.08 µmol/L, 0.19 nmol/L, and 2 µIU/mL, respectively (Siemens Medical Solution Diagnostics, CA, USA). The intra- and inter-assay coefficients of variation were 6.4% and 8.2% for A4, 4.9% and 8.8% for DHEAS, 5.8% and 8.6% for F, and 4.9% and 6.4% for insulin. Free T (fT) concentrations were measured using a free testosterone ELISA kit (GenWay Biotech Inc., CA, USA), with a sensivity of 0.006 pmol/L. The intra- and inter-assay coefficients of variation ranged from 5–10% and 8–12%, respectively. Levels of 17-OHP4 were verified using a coat-a-count radioimmunoassay (Siemens Health Care Diagnostics Inc., CA, USA) with a sensitivity of 0.67 nmol/L, and an inter- and intra-assay imprecision of 5.5% and 7.9%, respectively. After extraction, compound S was measured with an HPLC/RIA developed in-house by the Alvaro Center of Analysis and Clinical Research in Paraná, Brazil; the sensitivity was 0.3 nmol/L, and the inter-assay coefficients of variation were 5.3% and 10.8%. The level of 17-OHPE was measured with an HPLC/MS/MS (Labco Nous Advanced Special Diagnostics, SP, Brazil). The sensitivity was 0.033 nmol/L, and intra-and inter-assay coefficients of variation were between 10.4% and 12.9%, respectively. FAI was calculated as the T (nmol/L)/SHBG (nmol/L) × 100. Free estrogen index (FEI) was estimated as 100 × E2 pmol/L/272.14 × SHBG (9).

To identify the interference of possible outliers, the original data of each variable were initially submitted to the Grubbs test. After the exclusion of the outliers, the distribution was assessed using the Shapiro–Wilk test and those with a non-Gaussian distribution were transformed. The skewed data were linearized using a logarithmic or square transformation. Prior to the analysis, the data were back-transformed into the original units. The results are presented as the mean and standard deviation (s.d.). Differences between the three independent variables were assessed using one way analysis of variance, followed by the Tukey *post hoc* test and *F* test. The relationship between two variables was examined using the Pearson’s correlation coefficient (*r*). A stepwise multiple regression analysis was performed with 17-OHPE as the criterion variable and several anthropometric, endocrine and metabolic variables that presented a significant simple correlation coefficient with 17-OHPE as predictor variables. The Durbin–Watson test was used to verify correlation between residuals. All of the statistical procedures were performed with SPSS version 17 (SPSS). All tests were two-sided and *P* values <0.05 were considered statistically significant.

## Results

On an average, normal cycling control, normoandro­genemic PCOS and hyperandrogenemic PCOS patients were 30.35 ± 5.23, 28.71 ± 5.80, and 26.91 ± 5.07 years old, respectively (*P* < 0.001 for control vs hyperandrogenemic PCOS). Comparisons of anthropometric, metabolic and hormone parameters among groups are depicted in Table 1. Baseline concentrations of PRL, TSH and T4F were not different between control and PCOS groups. Progesterone, estradiol, cortisol, compound S and 17-OHP4 concentrations were not different between normal cycling women and PCOS subjects as a whole group. Hyperandrogenemic PCOS presented higher levels of insulin, C-peptide and of all androgens when compared with normoandrogenemic PCOS and control women. Normoandrogenemic PCOS presented higher levels of total T, 17-OHPE, and insulin compared with normal cycling women. All the markers of glucose metabolism were higher in both PCOS groups than in healthy cycling women. The precursor-androgen 17-OHPE also was higher in normoandrogenemic and hyperandrogenemic PCOS than in controls (*P* < 0.001).

**Table 1:** Comparisons of anthropometric metabolic and hormonal parameters among healthy controls, and normo- and hyperandrogenemic PCOS patients.

	**Healthy controls** (*n* = 91)		**Normoandrogenemic PCOS** (*n* = 46)		**Hyperandrogenemic PCOS** (*n* = 147)	
**Variable**	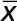	**s.d.**	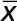	**s.d.**	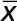	**s.d.**
Anthropometric						
Weight (kg)	61.96	11.11	74.18	19.89^a^	77.51	16.41^a^
BMI (kg/m^2^)	23.49	03.43	29.14	8.22	32.86	6.57^a^
Waist (cm)	73.23	8.12	86.30	16.32^a^	89.80	14.10^a^
WHR	0.74	0.05	0.80	0.07	0.82	0.08^b,c^
FM (kg)	29.17	5.88	35.72	8.65	39.37	7.70^a,d^
C Index	1.09	0.08	1.16	0.10	1.18	0.10^a^
VAI	1.17	0.90	2.15	2.06	2.63	2.03^c^
LAP	13.83	11.70	39.32	31.65	52.68	43.99^a^
Metabolic						
Glucose (nmol/L)	4.71	0.49	4.96	0.40^a^	5.08	0.62^a^
HOMA-IR	0.92	0.44	1.55	0.71^b^	2.26	1.41^c,d^
HOMA %B	103.12	44.81	142.38	49.26^a^	153.60	61.99^a^
HOMA-S	138.18	72.65	77.73	47.43^a^	65.37	44.89^a^
HbA1C (%)	4.89	0.52	5.44	0.90^a^	5.77	1.07^a^
TC (mmol/L)	4.26	0.69	4.57	0.86^a^	4.88	1.23^a^
HDL (mmol/L)	1.39	0.34	1.22	0.33	1.14	0.31^e^
VLDL-C (mmol/L)	0.37	0.18	0.51	0.32	0.77	0.44
TG (mmol/L)	0.93	0.46	1.40	0.40	1.58	0.96^e^
Hormonal						
Insulin (µmol/L)	50.1	24.7	89.0	47.5	118.2	74.5^b,c,d^
C-pep (nmol/L)	0.5	0.2	0.6	0.3	0.9	0.5^c,f^
P4 (nmol/L)	1.6	1.3	1.1	0.6	1.6	1.0
E2 (pmol/L)	168.9	82.7	170.5	64.9	188.5	73.4
FEI (%) pg/nmol)	0.3	0.1	0.4	0.1	0.8	0.3^d,c^
Testo T (nmol/L)	0.9	0.5	1.1	0.4	2.3	0.9^a,f^
Free T (pmol/dL)	2.0	0.4	2.0	0.2	6.0	0.1^f,c^
SHBG (nmol/L)	59.7	27.7	45.9	20.9	32.90	20.1^c,b,f^
FAI (%)	1.8	0.3	2.8	0.3	8.9	0.6^c,f^
DHEA (nmol/L)	18.8	13.0	15.3	6.7	21.8	11.3^d^
DHEAS (µmol/L)	4.1	1.8	3.7	1.5	5.5	2.4^f,c^
A4 (nmol/dL)	7.0	1.0	5.0	4.0	11.0	1.0^d,c^
F (nmol/L)	364.2	148.6	318.7	132.4	348.3	139.1
Compounds S (nmol/L)	6.8	2.6	6.3	3.5	7.4	5.0
17-OHP4 (nmol/L)	2.4	1.0	2.9	1.7	3.0	1.8^e^
17-OHPE (nmol/L)	4.4	3.2	5.6	4.3	7.7	5.9^c,d^

To convert from SI to conventional units, divide by the factors: glucose = 0.0555, insulin = 6.945, TC = 0.0259, TG = 0.0113, C-pep = 0.333, P4 = 3.18, E2 = 3.671, T = 0.0347, Free T = 0.0347, DHEA = 3.47, DHEAS = 2.71, A = 3.49, F = 25.27, compounds S = 28.86, 17-OHP4 = 3.03, 17-OHPE = 3.01.

a *P* < 0.001, control vs normo- and hyperandrogenemic; ^b^ *P* < 0.05, control vs normoandrogenemic;

c *P* < 0.001, control vs hyperandrogenemic; ^d^ *P* < 0.05, normoandrogenemic vs hyperandrogenemic;

e *P* = 0.05, control vs hyperandrogenemic; ^f^ *P* < 0.001, normoandrogenemic vs hyperandrogenemic.

In normal cycling controls, 17-OHPE was not correlated with any anthropometric or metabolic parameter but showed a negative correlation with compound S (*r* = −0.247, *P* = 0.025), and a positive correlation with FEI (*r* = 0.479, *P* = 0.028), and DHEAS (*r* = 0.248, *P* = 0.021). In a stepwise multiple repression, only DHEAS was retained in the model with an adjusted *R*^2^ = 0.127, standardized beta coefficient *B* = 0.357 (*t* = 2.752, *P* = 0.008). Therefore, in normal controls DHEAS levels accounted by 12.7% in the 17-OHPE variation (*F* = 7.571, *P* = 0.008); Durbin–Watson’s correlation between residues of 2.239.

In the normoandrogenemic PCOS group the influence of all tested variables on 17-OHPE concentrations was initially examined by simple correlation and further by stepwise multiple regression. In the simple correlation VAI (*r* = 0.365, *P* = 0.011), LAP (*r* = 0.318, *P* = 0.043), insulin (*r* = 0.414, *P* = 0.008), HOMA-IR (*r* = 0.408, 0.011) and cortisol (*r* = 0.311, *P* = 0.048) presented significant positive correlation with 17-OHPE concentrations. The stepwise multiple regression showed that only LAP with an adjusted *R*^2^ = 0.206, standardized beta coefficient *B* = 0.453, *t* = 2.878, *P* = 0.007 remained in the final model. Considering this model, variation of 17-OHPE accounted by this predictor was 20.6% (*R*^2^ adj = 0.181) and this model represents a good fit of the data (*F* = 8.283, *P* = 0.007). The Durbin–Watson’s correlation between residues was 1.590.

Taking in account the hyperandrogenemic PCOS patients, 17-OHPE presented significant correlation with several anthropometric, metabolic and hormonal parameters (Table 2). Even most of correlations had been weak or moderate, they were highly significant. The importance of these correlations was further tested using a stepwise multiple regression with 17-OHPE as the criterion variable and variables shown in Table 2 as predictors of the 17-OHPE variation. According to the model (Table 3), WHR and HDL-C accounted for 24.5% of 17-OHPE variability (*R*^2^ = 0.245) and this model showed to be a good fit of the data (*F* = 10.394, *P* < 0.001). In addition, the Durbin–Watson’s correlation between residuals was 1.909. The contribution of each variable, given by standardized coefficients, was as follows: WHR (*B* = −0.404, *t* = −3.986, *P* < 0.01), HDL-C (*B* = 0.055, *t* = 2.034, *P* = 0.046).

**Table 2:** Significant simple correlation between 17-OHPE concentrations and anthropometric, metabolic and hormonal parameters in polycystic ovary syndrome patients with hyperandrogenemia.

**Variable**	**Pearson’s coefficient correlation (*r*)**	***P***
Anthropometric		
WC (cm)	−0.365	0.001
BMI (kg/m^2^)	−0.197	0.021
WHR	−0.439	<0.001
FM (kg)	−0.276	0.011
C Index	−0.324	0.003
LAP	−0.269	0.003
VAI	−0.261	0.005
Metabolic		
HDL-C (mmol/L)	0.297	0.012
VLDL-C (mmol/L)	−0.185	0.041
TG (mmol/L)	−0.181	0.036
HOMA-IR	−0.217	0.025
HOMA %B	−0.282	0.009
HOMA-S	0.466	<0.001
Hormonal		
E2 (pmol/L)	−0.203	0.047
FEI (%) pg/nmol	−0.244	0.021
PRL (nmol/L)	0.172	0.047
SHBG (nmol/L)	0.393	0.004
A4 (nmol/L)	0.269	0.002
DHEA (nmol/L)	0.322	0.001
C-pep (nmol/L)	−0.370	0.001

**Table 3:** Stepwise multiple regression between 17 hydroxypregnenolone and significant predictors found using simple linear correlation in PCOS with hyperandrogenemia.

**Model**	***R***	***R*^2^**	**Adjusted** ***R*^2^**	**s.d. of estimate**	***F***	**Durbin–Watson**	***P* value**
1	0.443^a^	0.196	0.184	2.2687	15.885		<0.001^a^
2	0.495^b^	0.245	0.222	2.1643	10.394	1.909	<0.001^b^

a Predictor: 17-OHPE nmol/L (Constant)*, WHR;

b predictor: 17-OHPE nmol/L (Constant), WHR, HDL-C;

* 17-OHPE, criterion variable.

## Discussion

The present study was designed to examine the potential connections of 17-OHPE, a product of ovarian theca, and fasciculate and reticularis adrenal cells under P450_C_17α and 3β-HSD II enzyme expressions with anthropometric, metabolic and hormonal parameters in normal controls and in PCOS subjects with high androgens in blood. In summary, it was demonstrated that PCOS patients, either with normal or high androgen levels, presented higher 17-OHPE concentrations than controls. 17-OHPE presents different correlations with anthropometric and metabolic parameters in PCOS patients, according to their androgen profile. The anthropometric abnormalities found in hyperandrogenemic PCOS, are in agreement with the current knowledge emphasizing a complex crosstalk between adipocyte products and ovarian/adrenal steroidogenesis. The relationship between visceral adiposity and hyperandrogenemia is complex. This matter is discussed considering several anthropometric parameters commonly related with adipocyte distribution and adrenal androgen precursors.

As novelty, the present study provided new knowledge regarding 17-OHPE relationship with several anthropometric, hormonal and biochemical parameters in PCOS patients, mainly in those with higher concentrations of androgens. However, the clinical implications of these findings await for prospective studies designed to attend this purpose. Even the current study has used clear definition for PCOS and biochemical hyperandrogenemia, a few possible limitations need to be considered when interpreting the present findings. The criteria used to exclude the non-classic enzyme deficiencies may not be universally adopted. Blood concentrations of 17-OHPE is also not widely measured in PCOS in most chemistry laboratory and data for comparisons are scarce, limiting a deeper discussion. The concern with imprecise androgen measurement seems not to be justified because comparisons of the results between the assays used in the current study for T, 17-OHPE, 17-OHP4, and compound S and liquid chromatography tandem mass spectrometry have shown good agreement between the methods (28, 29).

Some adipocyte-derived products may induce the transcription of the StAR promoter protein and modulate steroidogenic enzyme activities (30, 31). In contrast, DHEA has shown anti-adipogenic activity on adipocyte cells with capability of improving adipocyte insulin sensitivity and improve adipokine profile (32). In the present study, besides correlating with DHEA, the concentrations of 17-OHPE showed different relationship with several anthropometric and metabolic parameters in normo and hyperandrogenemic PCOS patients. Most markers of adipocyte dysfunction/distribution (LAP, VAI, BMI, WHR, WC) were positively correlated with 17-OHPE in PCOS when the androgen levels were normal. Otherwise, these parameters were negatively correlated with 17-OHPE when PCOS patients had increased androgen concentrations. Therefore, it seems that adipocytes distribution/number influence the steroidogenesis in different ways in PCOS, depending on the baseline androgen levels.

The positive simple correlations between 17-OHPE concentration and VAI, LAP, HOMA-IR, insulin and cortisol have not been previously described in normoandrogenemic PCOS. The persistent positive correlation between 17-OHPE and LAP after multivariate regression demonstrated that part of the 17-OHPE variability in these patients is associated with the WC and TG levels. In addition VAI, which is a composite of WC, TG, and HDL-C, has been clinically used as indicator of adipose dysfunction and cardiometabolic risk (33). Therefore, 17-OHPE levels in PCOS patients with normal levels of androgen are associated with markers of abnormal fat distribution and its measurement could have clinical utility in these patients. It is known that adiponectin, a product of adipocyte with receptors in ovary (34) and adrenal (35) cells, increases StAR protein and the expression of some steroidogenic enzymes (36). Furthermore, the adipose tissue product leptin may also inhibit 3β-HSD II activity and increase 17-OHPE levels (37, 38). The positive correlation between 17-OHPE and markers of dysglycemia found in the present study is in agreement with the previous knowledge that PCOS patients with insulin resistant have adipose dysfunction, despite the levels of androgens in blood.

LAP, composed by WC and TG, used as an indicator of insulin resistance, has already been associated with increased BMI, fasting glucose and fasting insulin in PCOS (39, 40). It was also shown that adipocyte-derived products have a stimulating effect on DHEA and cortisol release by adrenal cells (41). We have previously shown a negative correlation between WC and 17, 20 lyase activity in the ∆5 pathway in normoandrogemic PCOS subjects (9). Different from the observed in normoandrogenemic PCOS, the significant negative simple correlation observed between most anthropometric parameters and 17-OHPE in the current study indicated that abnormal adipocyte mass distribution exercise a possible role in the control of 17-OHPE secretion in PCOS patients with high androgen in blood. Previously, we, and others, have demonstrated higher BMI, WC, C index, WHR and FM in hyperandrogenemic PCOS (9). The significant negative correlation between LAP, VAI, TG, HOMA-IR, HOMA %B and the levels of 17-OHPE in PCOS with high androgens is then the opposite of that observed in normoandrogenemic PCOS. WC was shown to be positively correlated with 17, 20 lyase activity in the ∆5 pathway (4) and BMI has been shown to be positively correlated with leptin and insulin in PCOS (42). In contrast, WHR was found to be negatively correlated with leptin in PCOS (42). The impact of adipocyte products on specific steroidogenic enzyme activity is clearly a field of new researches.

In hyperandrogenemic PCOS, 17,20 lyase effectivity is less in the ovaries than in the adrenal (43). A negative association of 17-OHPE and BMI demonstrated in the present study was already found in pubertal girls (44), but evidences of clinical implications regarding 17-OHPE association with anthropometric parameters are very limited at this time. Relationship of anthropometric parameters with accumulus of adipose tissue and other adrenal precursor-androgen such as DHEA have already been reported. Several studies have examined the relationship between obesity or WHR and DHEA/DHEAS in non-PCOS subjects many of them reported negative correlations, suggesting that higher circulating DHEA is associated with lower body fat accumulation (45). However, some studies referred positive correlation between this androgen precursor and WHR (46). The reasons for these discrepancies are yet unclear. Leptin, which is increased in obesity, activates 17-hydroxylase in the ∆5 pathway (30). Moreover, insulin, commonly high in hyperandrogenemic PCOS, was also demonstrated to activate 17-hydroxylase enzyme activity (30). Unfortunately, adipokines were not measured in the current study to expand the current knowledge.

It was demonstrated that the activities of 17,20 lyase and 3β-HSD II can be affected by adipose tissue, fat distribution and glucose/insulin balance (30, 31, 32, 33, 34, 44). Putative mechanisms may involve growth factors, insulin, cytokines, free fatty acids and inflammatory markers (44). Additionally, it can be hypothesized that intra-adrenal factors, or androgen precursor in the inner zone reticularis, may operate in hyperandrogenemic PCOS patients, explaining their correlation with 17-OHPE concentrations (47). Increase in DHEAS levels has been associated with increased BMI and WHR in PCOS patients (48, 49, 50). Otherwise, DHEAS has shown a negative correlation with BMI in non-PCOS subjects (50, 51, 52). These discrepant results are not clear at this time. Further, in previous study, a negative relationship between DHEA levels and cardiovascular risk in women suggested that higher levels of certain adrenal precursor-androgen may be protective against metabolic and cardiovascular disease (49). A positive correlation between A4 and BMI was also reported in PCOS patients (51, 53). In addition, 17-OHPE was shown to be positively correlated with DHEA and A4 in the current study.

The negative correlations between 17-OHPE and most markers of insulin resistance, and the positive correlations with HDL-C, and HOMA-S indicated that this androgen intermediate may be used as a marker of insulin tolerance in hyperandrogenemic PCOS patients, and could indicate a certain protection against cardiovascular disease in this group. Basal and stimulated levels of 17-OHPE, and DHEA, were found to be positively associated with the ability of glucose to control its own production in PCOS women, but not in healthy subjects (54). In fact, an early study demonstrated that hyperglycemia in diabetic-PCOS patients was associated with the degree of adrenal hypersecretion (55). Otherwise, one single study has not demonstrated any correlation between APA secretion and insulin resistance in PCOS patients (56). However, the use of metformin was shown to decrease ∆5 17-OHPE and 17-OHP4, indicating that increased levels of adrenal androgens, including 17-OHPE, are negatively associated with insulin sensitivity markers (57).

The negative correlations between 17-OHPE and E2 levels and FEI and positive correlation between 17-OHPE and SHBG seen in hyperandrogenemic PCOS in the current study indicated that estrogen can modulate steroidogenic pathways in these patients. In addition, chronic hyperestrogenism has been shown to modulate adrenal responsiveness in PCOS subjects, particularly by modulating 17,20 lyase activity (58). In contrast, E2 may decrease the 3β-HSD II activity and enhances ∆5 adrenal androgen synthesis (43), including 17-OHPE. Moreover, the activity of 17,20 lyase in the ∆5 pathway was found to be lower in both normo- and hyperandrogenemic PCOS, explaining the increased levels of 17-OHPE in PCOS (2). Furthermore, the most frequent reported finding in PCOS is a greater 17-hydroxylase activity in the ∆5 pathway (59) and 17, 20 lyase activity has shown to be higher only in the ∆4 pathway in hyperandrogenemic PCOS patients (2).

## Conclusion

17-OHPE exhibited different relationships with anthropometric, biochemical and hormonal parameters in PCOS patients, depending on the androgen levels. In PCOS subjects with high androgen concentrations, 17-OHPE was negatively associated with most anthropometric parameters, particularly with those used as markers of adipose tissue dysfunction and frequently used as predictors of cardiovascular disease risk. Furthermore, the positive correlation of 17-OHPE with HDL-C and HOMA-S suggests that hyperandrogenemic PCOS patients with high levels of 17-OHPE could have a certain protection against cardiovascular disease. Future studies are required to evaluate the clinical implication of these novel findings.

## Declaration of interest

The authors declare that there is no conflict of interest that could be perceived as prejudicing the impartiality of the research reported.

## Funding

This research did not received any specific grant from any funding agency in the public, commercial or not-for-profit sector.
